# Antagonistic PCP Signaling Pathways in the developing *Drosophila* eye

**DOI:** 10.1038/s41598-018-24053-3

**Published:** 2018-04-10

**Authors:** Vladimir L. Katanaev, Diane Egger-Adam, Andrew Tomlinson

**Affiliations:** 1 Department of Genetics and Development, College of Physicians and Surgeons, Columbia University, 701 West 168th St #1120, New York, NY 10032 USA; 2 Department of Biology, University of Konstanz, Universitätsstrasse, 10, Box M643, 78467 Konstanz, Germany; 3 Department of Pharmacology and Toxicology, University of Lausanne, Rue du Bugnon 27, CH-1005 Lausanne, Switzerland; 4 Department of Genetics and Development, College of Physicians and Surgeons, Columbia University, Jerome L. Greene Science Center, MC9892, Level 9 Room 028, 3227 Broadway, New York, NY 10027 USA

## Abstract

In Planar cell polarity (PCP), cells coordinately polarize their cytoskeletons within the plane of the epithelium in which they lie. In most insect epithelia this is indicated by the coordinated projections of the hairs secreted by the ectodermal cells. PCP of this form has been effectively studied in Drosophila, but it has proven difficult to achieve an integrated description of the roles played by the various proteins. In the insect eye, PCP is not evident as the polarization of individual cells, but as the asymmetric arrangements of the cells of the ommatidia. This different form of PCP allows different studies to be performed, and using this system we have detected the action of two antagonistic signaling pathways. Even though antagonistic, the two pathways synergize and cooperate to ensure that the correct arrangement of the cells is achieved. The cooperative use of antagonistic signaling pathways occurs in the polarization of chemotacting cells, and we discuss the possibility that a similar molecular principle may underlie PCP.

## Introduction

In planar cell polarity (PCP) cells coordinately polarize their cytoskeletons within the plane of the epithelium in which they lie. When such epithelia are decorated with hairs or bristles, the coordinated polarizations are visibly manifest as the uniform orientation of these structures over extensive tracts. Beginning with the work of Gubb and Garcia Bellido^1^, many genetic studies have been performed and many key proteins identified. Genetic analysis of the system has been hampered by the fact that loss-of-function phenotypes often appear similar to those of the gain-of-function; both manifesting as the disruption of the cellular polarities. The fly eye displays a strikingly different manifestation of PCP; here it is the geometric arrangement of a group of cells rather than the direction of hairs projecting from individual cells. This different type of PCP is amenable to different technical approaches, and loss-of-function effects can be readily distinguished from those of gain-of-function.

**Figure 1:**
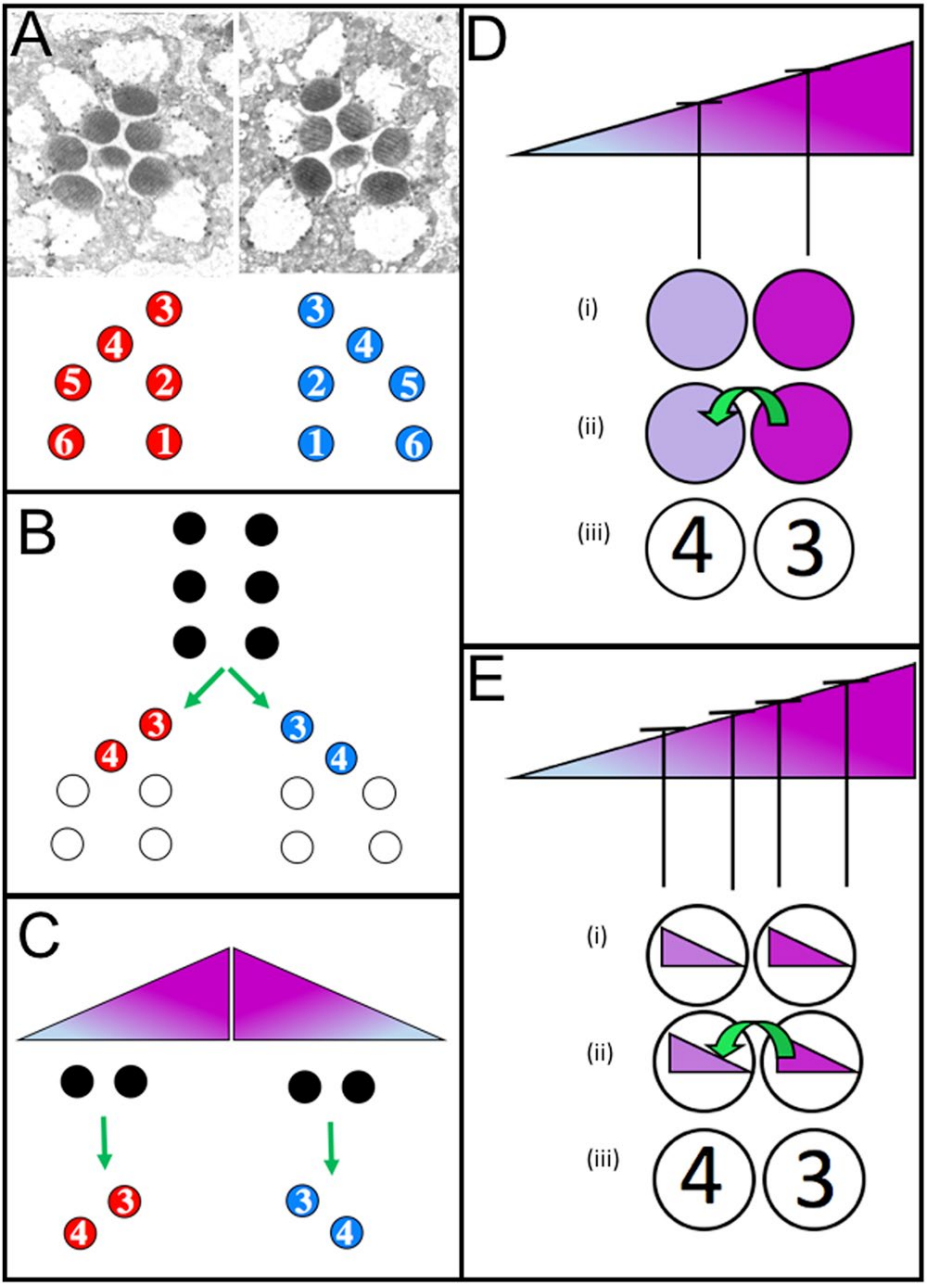
Features of PCP in the fly eye (**A**). Ommatidia occur in two mirror-symmetric (chiral) shapes that we color-code as red and blue. (**B**) Ommatidia begin life as symmetric structures (color-coded as black), and then adopt the red or blue shape. It is the cells in the R3/4 positions that decide which shape ommatidia will form. If the cell on the left becomes R3 (and the other R4) then the blue shape is formed, but if the cell on the right becomes R3 (and the other R4) then the red shape results. (**C**) The polarizing gradient directs the R3/4 decisions, with the cell higher in the gradient being directed to become R3 (and the other R4). (**D**) In the scalar model the cells in the R3/4 positions meter the ambient gradient concentration using Fz and (i) each establishes a commensurate internal activity, (ii) Fz activity is higher in the pre-R3 which directs the N/Dl interaction (green arrow) between the two cells and (iii) the cell with the Fz higher activity is directed to become R3. (**E**) In the vector model, the cells meter the change of the gradient across their diameters. (i) In this model Fz activity is repressed by the polarizing information (see ref.^8^ for a full description) and Fz activity gradients are established in the cells in a mirror symmetric manner to the polarizing gradient. (ii) At the point of contact of the R3/4 pair, the Fz activity is higher in the pre-R3 which directs the N/Dl interaction (green arrow), and (iii) this cell becomes R3 (and the other R4).

The Drosophila eye is made from many hundred subunit ommatidia, each of which adopts a specific asymmetric (chiral) cellular organization (Fig. 1A)^2^. Chirality is evident in many ommatidial features - the asymmetric arrangements of the photoreceptors; the position of the R8 cell; the projection of the cone cells through the photoreceptor grouping; and the positions of the sensory bristles. All these asymmetries correlate in any one chiral form, and in this work we focus on the most salient chiral feature - the asymmetric trapezoidal arrangement of the six outer-photoreceptors (R1–6) (Fig. 1A). The asymmetric shapes occur into two chiral forms (which we color-code as red and blue), the one being the mirror image of the other, and the only difference between them being the asymmetric positions of the R3 and R4 (R3/4) cells. In any adult eye, all ommatidia in the dorsal half are of one chiral form while those in the ventral region are all of the other. When ommatidia are first formed they are symmetric structures^3^ (achiral; which we color-code as black), and as they develop they acquire asymmetry, adopting the red or blue shape. This choice is mediated entirely through the R3/4 pair of cells, and as one adopts the R3 position so the other becomes R4 (and vice versa; Fig. 1B)). The R3/4 asymmetry is directed by a Fat (Ft – a large Cadherin-like transmembrane protein) activity gradient present within the developing retina that inverts at the D/V midline (the equator) (Fig. 1C)^4^. Ommatidia detecting the gradient falling off to left become red shapes, while those detecting the decline to the right become blue. The Ft activity information is relayed into the cells (by an unknown mechanism) through the action of the plasma membrane receptor Frizzled (Fz)^5^, and as a result a Fz activity disparity results in the R3/4 pair. This Fz activity differential is then relayed to a Notch/Delta (N/Dl) interaction mechanism that occurs between the two cells which directs one cell to become R3 and the other R4^6–8^. This N/Dl lateral interaction is similar to that which occurs between two cells of developing nematode vulva in the specification of the anchor cell. Here, two equipotent cells laterally interact using the nematode equivalents of N and Dl (Lin12 and Lag2 respectively). Initially the N signaling between the two cells is roughly equivalent, but through an amplifying feedback mechanism, a slight disparity between the two becomes rapidly increased and consolidated, resulting in one cell with high N activity and the other low^9^. In R3/4 cells, the N/Dl interaction is biased by the input from the polarity information; here, the cell with the higher Fz activity ends with low N activity (the R3 fate) and the other with high (R4).

Two models emerged to explain how the R3/4 disparity was established through differential Fz activity^6–8^. One (the scalar model) envisages that each cell of the R3/4 pair “reads” its local position in the gradient and establishes a commensurate Fz activity (Fig. 1D). The N/Dl interaction mechanism then meters those activities, detects that one cell has more Fz activity than the other (Fig. 1D, green arrow) and specifies that as the R3 (and the other as R4). The other model^8^ (the vector model) proposes that cell polarization lies at the heart of the R3/4 asymmetry mechanism, and that each cell relays the Fz activity across its diameter into an intracellular gradient, high at one end and lower at the other (Fig. 1E). As a result, both R3/4 s have a graded Fz activity along their D/V axes, but at the key position where the two cells contact and where the N/Dl interaction occurs, one cell has the high end of its gradient while the other has the low end. It is this local disparity in the polarized information that biases the N/Dl interaction (Fig. 1E, green arrow) and drives the formation of the appropriate chiral shape. Two observations suggest that the vector model is correct. First, many of the proteins that are directly involved in PCP in the other epithelia (in which cell polarization clearly occurs) also act in the eye mechanism, and one would expect them to execute similar molecular functions in both tissue types. Second, many of the proteins adopt asymmetric localizations within the cells of standard epithelia that correlate with their actions in the polarization phenomenon^10–13^, and asymmetric accumulations have also been observed in the R3/4 cells^14^.

In this paper, we use the R3/4 fate readouts to infer the actions of two distinct pathways, one of which promotes the ability of a cell to be specified as R3 (while the other cell becomes R4), and another that promotes the R4 fate (while the other cell becomes R3). Thus, we define two antagonistic pathways at play in eye PCP. However, although the pathways appear antagonistic (as defined by their R3/4-promoting activities) we find that they act cooperatively to ensure that the ommatidia make the correct R3/4 choice. We consider the process to be in principle similar to the opposing activity gradients that polarize the cytoskeletons of chemotacting cells, and we discuss the similarities and differences between the two.

## Materials and Methods

### Genetics

The transgenes were: *UAS-Go*^*wild type*^; *UAS-Go*^*GTP*^; *UAS-Go*^*GDP*^ ^15^; *UAS-Gγ1*^16^; *UAS-Gβ13F*; *UAS-Gβ5* ^17^; *tub > y*^+^ > *Gal4, UAS-w*^+^ (G. Struhl); Mutant lines were: *Go*^007^ ^18^, alone or recombined with *frt42D*; *Go*^0611^ ^15^ alone or recombined with *frt42D, pwn*; *Gγ*1^*N159*^ ^16^ recombined with *frt42D*; *RhoA*^*72BH*^ and *RhoA*^*72M1*^^19^ recombined with *frt42D*; *lola*^*5D2*^ ^20^; *dsh*^1^ ^21^; *dsh*^*V26*^ ^22^ recombined with *frt101*; *fz*^*−/−*^ animals were *fz*^*H51*^/*fz*^*KD4A*^ ^23^; *MS1096-Gal4* ^24^ was used directly or recombined with *dsh*^*1*^. Mitotic clones were induced using *hsp70-flp*^25^ by heat-shock (1 h 37 °C). *eye-flp*^26^ was used to generate overexpression clones marked with appearance of *w*^+^ expression. A new allele of Go (*Go*^19^) was generated by imprecise excision of a G-oα47A^CA06658^ ^27^, a *w*^+^ P-element insertion in non-coding sequence of the gene. Technical problems have prevented us from defining the exact nature of the *Go*^19^ lesion, but it behaves as a hypomorphic allele; the deletion removes at least a portion of the *Go* gene, and Go protein levels are dramatically reduced in clones (not shown).

### Histology

Eyes were processed for sectioning and analysis following^28^. Adult wings were fixed in GMM. Analysis of the multiple wing hair and asymmetric cell division phenotypes was performed as described^15,17^.

### Statistical analysis

Was performed using the unpaired T-test treating results from individual animals as separate entries. Statistically significant differences (mean ± sem) are denoted with “**” (P value < 0.05), “***” (P value < 0.005), and “****” (P value < 0.0001).

### Data availability

The authors affirm that they will make all materials, data and protocols freely available on request to the corresponding author.

## Results

### Dishevelled preferentially promotes the R3 fate

**Figure 2:**
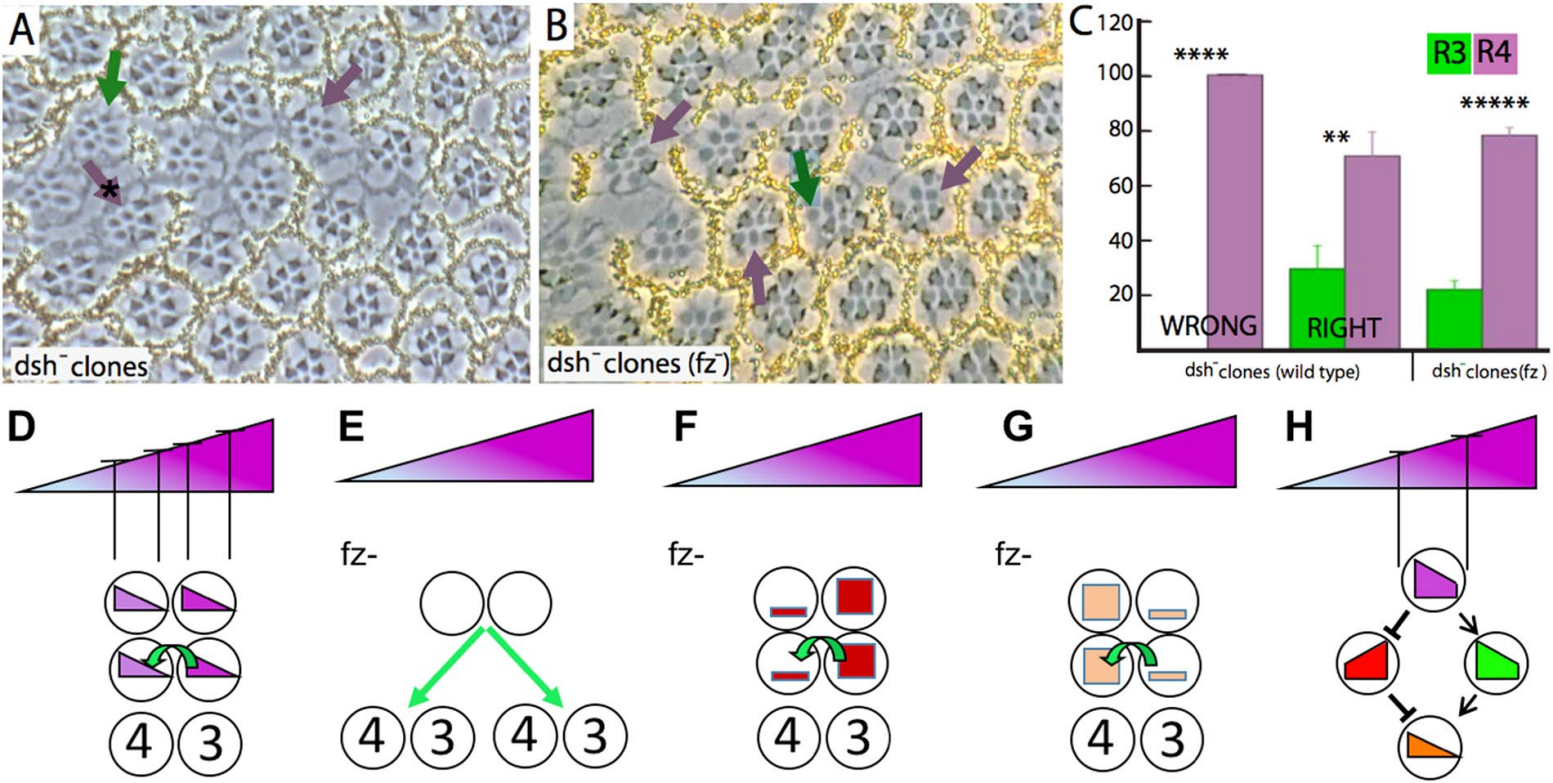
Dsh promotes the R3 fate (**A**). Clonal analysis with *dsh*^*V26*^. Mutant cells are marked by the absence of pigment. When the ommatidia adopt the wrong chiral shape, the R4 cell is invariably mutant (purple arrow). When the ommatidia adopt the correct chiral shape, the R4 cell may be mutant (purple arrow with asterisk), or the R3 cell may be mutant (green arrow). (**B**) *dsh*^*V26*^ clonal analysis in a *fz*^*−*^ mutant background. Purple arrows indicate ommatidia in which R4 is mutant, and the green arrow point to an ommatidium in which R3 is mutant. (**C**) Histogram displaying the frequencies of *dsh*^*V26*^cells in the R3 and R4 positions of mosaic R3/4 pairs in *wild type* and *fz*^*−*^ backgrounds. 5 eyes were analyzed for each background, with the overall number of R3/R4 mosaic ommatidia scored being 107 for the *wild type* background and 208 for the *fz*^*−*^ background. (**D**–**H**) Schematic models of how R3/4 fates may be specified. (**D**) In the standard vector model the cells use Fz to decode the external gradient to direct the R3/4 fates. (**E**) In the absence of Fz, the cells are blind to the polarizing gradient and the N/Dl interactions resolve the fates randomly. (**F**) In the absence of Fz, if one of the cells has more of a protein that promotes the R3 fate, that cell will be specified as R3. (**G**) In the absence of Fz, if one of the cells has more of a protein that promotes the R4 fate, that cell will be specified as R4. (**H**) The Fz internal gradient (purple trapezoid) in each of the R3/4 cells may regulate two subservient molecules; positively regulated (green trapezoid) and the other negatively (red trapezoid). The two activities are antagonistic and their combined effects generate a “sharpened” polarizing gradient (orange triangle).

Fz acts both as a Wnt receptor and as the receptor for the polarizing information in PCP^1,29,30^, and in both these functions the cytosolic phosphoprotein Dishevelled (Dsh) appears as an immediate Fz transducer^21^. Dsh likely regulates different effectors in the two pathways, and is therefore a bifunctional transducer of Fz activity information. In this section we examine the role played by Dsh in eye PCP. Anecdotal accounts have suggested that Dsh acts in the R3/4 fate decisions to promote R3, but no study had been published in this regard. We therefore performed a *dsh* clonal analysis (using a *dsh* null allele) and examined the chiral shapes of ommatidia that formed when one of the R3/4 pair was mutant for *dsh*. In each hemisphere of an eye, the ommatidia are all of the same chiral shape, and when scoring the *dsh* R3/4 mosaics we not only asked whether the mutant cell was of the R3 or R4 type, but whether the shape of the ommatidium was correct for the part of the eye in which it was located. When an ommatidium was mosaic in the R3/4 pair and was of the incorrect shape (53 ommatidia), the R4 cell was invariably mutant (and the R3 wild type) (Fig. 2A,C). Since these were of the wrong chiral shape, this suggests that removal of Dsh from the cell normally destined to be R3 (the pre-R3) can redirect the cell to the R4 fate (with the pre-R4 becoming R3). However, the presence of a *dsh*^*−*^ pre-R3 in an R3/4 mosaic did not invariably lead to the cell fate transformation; of those R3/4 mosaics making the correct choice (54 ommatidia) 31% had a *dsh*^*−*^ mutant R3 (Fig. 2A,C). This contrasts strikingly with *fz*^−^ R3/4 mosaics in which loss of Fz from one of the pair invariably leads to that cell becoming R4 and the other R3, regardless of whether the shape formed is correct or incorrect^8,31^. Thus Dsh appears similar to Fz in that it promotes the R3 fate, but it does so with a lesser potency.

We were concerned here with two potential experimental artifacts. First, although the *dsh*^*−*^ clones were induced in the first larval instar, perduring *dsh*^*−*^ gene products may have engendered an inappropriately weak phenotype. To address this concern we induced *dsh*^*−*^ clones at the blastoderm stage, and confirmed the presence of the correctly shaped ommatidia in which R3 is *dsh*^*−*^ mutant and R4 is wild type (not shown). Thus, perdurance appears as an unlikely explanation. Second, wild type tissue lying immediately equatorial (towards the midline) of *dsh*^*−*^ clones frequently show disturbed chiral shapes, resulting from the role played by Dsh in transducing Wg signaling in the eye^32^. Hence, we assessed whether the correctly shaped ommatidia in which R3 is *dsh*^*−*^ mutant (and R4 is wild type) were found in the equatorial region of the clones. Rather, they were found in all positions where R3/4 mosaicism occurred (the green arrow in Fig. 2A indicates such an ommatidium in the polar region of a clone), suggesting that the non-autonomous effects of *dsh*^*−*^clones were not relevant to these analyses.

We therefore infer that only a portion of the Fz polarization information is relayed by Dsh, which suggests the action of another Fz transducer.

#### *dsh*^*−*^ clones in a *fz*^*−*^ mutant background

The N/Dl interaction mechanism that specifies the R3/4 fates is remarkably sensitive, and can detect and amplify even minor activity differences between the cells. This was shown when Fz was removed from the eye, and a 2/3 *N* gene dosage was imposed on the R3/4 precursors. Even though the difference between the cells was only a single copy of the *N* gene, the cell with the extra copy invariably became R4 (and the other R3)^8^. When we repeated the *dsh* mosaic analysis in a *fz* mutant background we expected that the Dsh activity bias between the two cells would be reliably amplified by the N/Dl mechanism, and the *dsh*^*−*^ cell would invariably be specified as R4 (and the other as R3). This is not what we observed. Rather, we detected an incomplete bias for the *dsh*^*−*^ cell to become R4 (and the other R3), as we had observed in the wild type background (Fig. 2B,C). Before discussing the significance of this result, we need first to consider some experimental aspects. In the vector model, each cell polarizes in response to the Fz activity gradient over its diameter, and the resulting activity differentials at the point of interaction between the R3/4 cells direct the bias of the N/Dl interactions (Fig. 2D). But, in a *fz* mutant, the cells are blind to the external gradient; there is no activity differential between the two cells and the N/Dl interaction resolves the R3/4 fates randomly (Fig. 2E). When we perform mosaic analysis in the *fz* mutant background we establish a gene expression differential between the two cells, and determine whether that differential promotes the mutant cell to the R3 or R4 fates (Fig. 2F,G). Thus, in this experiment we do not envisage polarized information in the cells (as occurs in wild type), but rather see the cells with blanket levels of gene product expression across their diameters in a similar manner to the scalar model (Figs 1D, 2F,G). With these experimental conditions in mind, we now review the *dsh* mosaic analysis in the *fz* mutant background and consider two different explanations. First, Fz may be required for Dsh activity, and a Dsh protein level disparity between the two cells can only weakly influence the R3/4 decision in its absence. The second model posits another transducer that antagonizes Dsh function and is repressed by Fz activity. This activity would be expressed in a graded manner in a mirror image fashion to the Dsh activity gradient (Fig. 2H). In this model, since Fz represses the activity of this other transducer, then in the *fz* mutant background its activity will be high (de-repressed) and will buffer the effects of Dsh activity differential between the R3/4 precursors. Although at this point we cannot distinguish between these two models, our results below argue for the presence of a transduction pathway that antagonizes the role played by Dsh.

### Elements of a trimeric G protein complex preferentially promote the R4 fate

Heptahelical proteins are often referred to as G protein-coupled receptors because of their propensity to signal through trimeric G proteins. The trimeric complex consists of an α-subunit and a βγ dimer. In the resting condition, the α-subunit is bound to GDP and associates with βγ. The activated receptor functions as a nucleotide exchange factor, replacing GDP on the α-subunit with GTP. In the GTP-bound condition, the trimeric complex dissociates into the α and βγ elements, both of which are then free to engage downstream effector molecules. Previously, we implicated Go, a trimeric Gα-subunit, in Fz signal transduction, both in Wnt signaling and in PCP^15,33^ We were, however, unable to show a role for Go in eye PCP. Here using the *f*z^*−*^ mutant background we uncover roles for Go and βγ subunits in promoting the R4 fate.

#### *Go* clones in a *f*z^*−*^ mutant background

**Figure 3:**
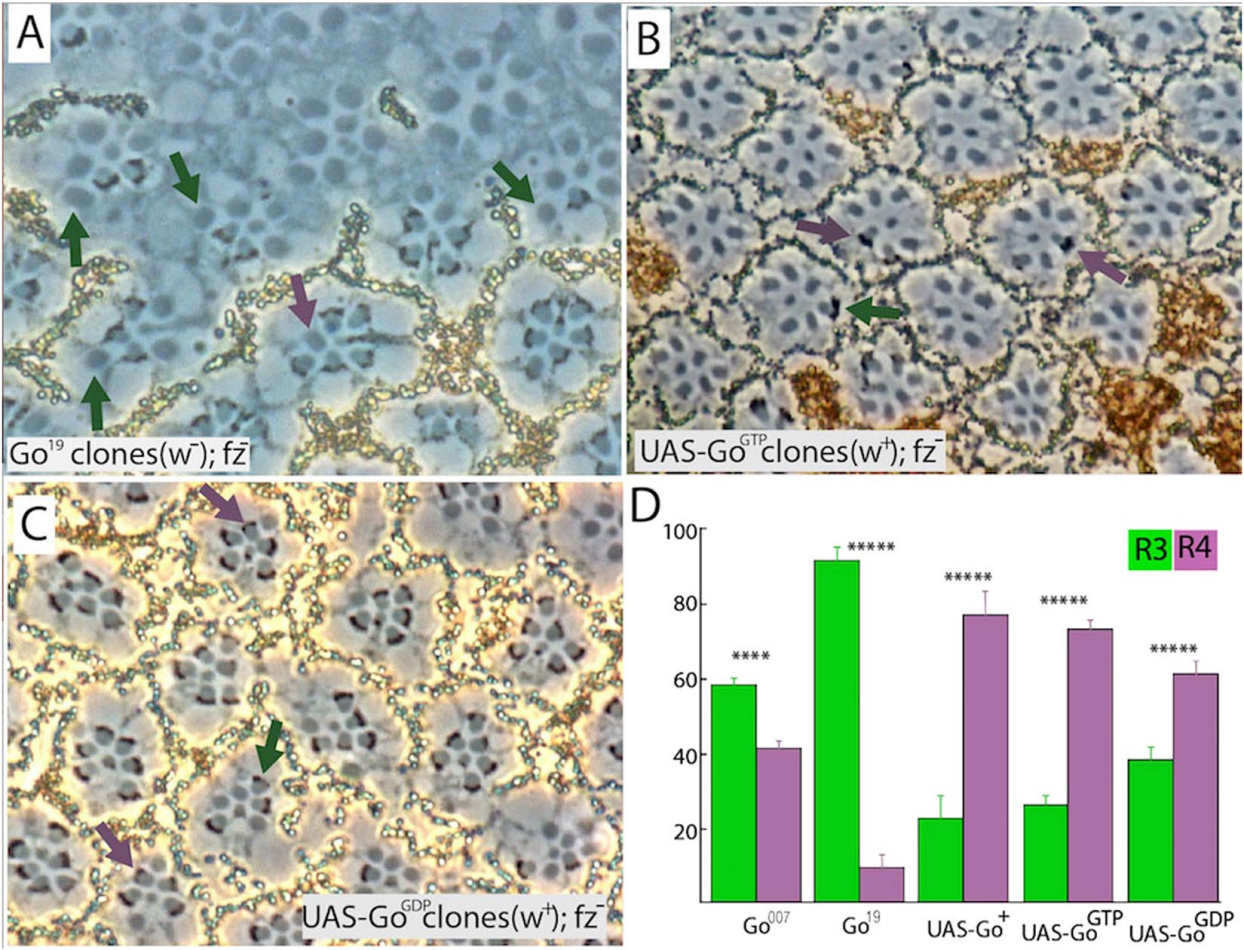
*Go* mosaic analysis in a *fz*^*−*^ mutant background. (**A**) *Go*^19^ clones (labeled by the absence of pigment) in a *fz*^*−*^ mutant background. The mutation affects the structure of the rhabdomeres, but this does not prevent scoring of the chiral shapes. In mosaic R3/4 pairs the cell mutant for Go is preferentially specified as R3 (green arrows), and less frequently specified as R4 (purple arrow). (**B**,**C**) When Go^GTP^ or Go^GDP^ are overexpressed (marked by the presence of pigment) in a *fz*^*−*^ mutant background and the R3/4 pair are mosaic the R4 fate is preferentially induced (purple arrows) over the R3 fate (green arrow). (**D**) Histogram summarizing the relative ratios of the R3/4 fates in R3/4 mosaics in the *fz*^*−*^ mutant background. ≥10 eyes were analyzed for each genotype, with the overall number of R3/R4 mosaic ommatidia scored ranging from 180 (*UAS-Go* wild-type) to 555 (*Go*^007^).

As we were performing this work, we became aware that the *Go*^007^ chromosome^18^ used in our previous studies^15^ carried other mutations. We therefore generated a new allele (*Go*^19^) on an independent chromosome to control for any genetic background effects (see Methods). When clones of *Go*^19^ or *Go*^007^ were induced in an otherwise wild type background, only infrequent effects on PCP were observed, but in a *f*z^*−*^ background when one of the R3/4 pair was mutant, that cell was preferentially found to be the R3 (Fig. 3A,D). Since reduction of *Go* causes a cell to adopt the R3 fate, we infer that normal Go function is to promote R4; the opposite to Dsh. To test further the role of Go in the R3/4 decisions, we assayed the effects in R3/4 mosaics of overexpression of three different forms of Go (Go^wild type^, Go^GTP^, and Go^GDP^). In otherwise wild type eyes the expression of these proteins showed minimal chirality effects, but in the *f*z^*−*^ mutant background all three preferentially promoted the R4 fate in R3/4 mosaics (Fig. 3B–D). Naively, we expected Go^GTP^ (the activated form) and Go^GDP^ (the dominant negative form) to promote opposite R3/4 fates, and the reasons why both promoted the R4 fate is addressed in the Discussion.

#### Gγ

When a trimeric G protein is activated (becomes GTP-bound) it releases its βγ moiety which may then engage downstream effectors. In this and the following section we express potential members of the βγ complex, singly or in combination, to determine whether effects can be detected on R3/4 fates.

**Figure 4:**
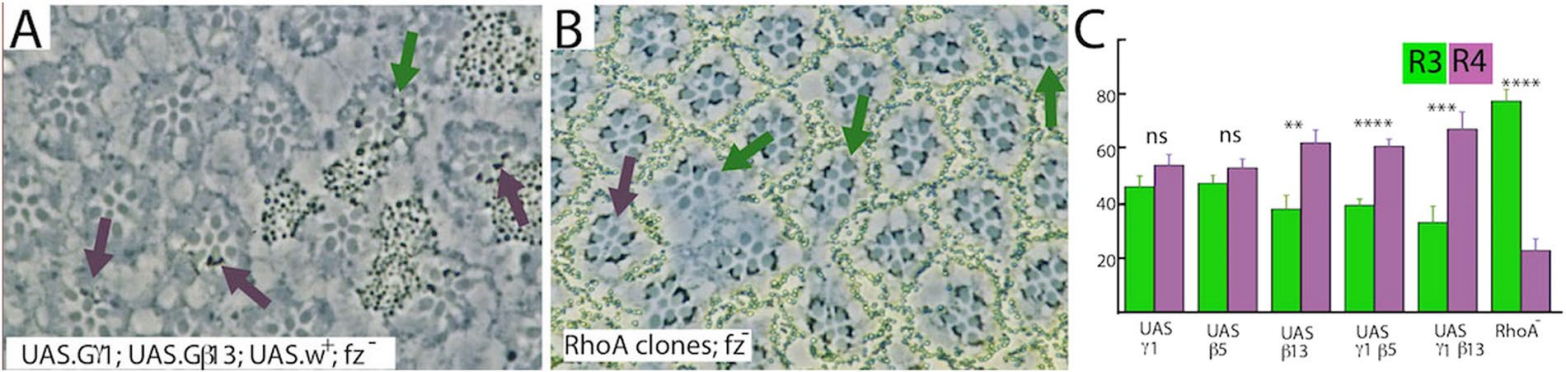
Mosaic analysis of *Gβ*, *Gγ* and *RhoA*. (**A**) Mosaic analysis of co-overexpression of G*γ*1 and G*β*13 in *fz*^*−*^ eyes. When the R3/4 pair are mosaic, co-overexpression of the two components, marked by the presence of the *w*^+^ transgene (pigment), preferentially induces the formation of R4 (purple arrows) over R3 (green arrow). (**B**) *RhoA*^*−*^ mosaics in *fz*^*−*^ eyes. When the R3/4 pair are mosaic the *rhoA* mutant cell (absence of pigment) preferentially promotes the R3 fate (green arrows) over the R4 fate (purple arrow). (**C**) Histogram plotting the relative ratios of R3/4 fates in R3/4 mosaic pairs when *Gβ* and *Gγ* genes are overexpressed, singly and jointly, and when *RhoA*^*−*^ clones are induced, in a *fz*^*−*^ background. For each genotype, ≥10 eyes were examined and ≥100 R3/4 mosaic ommatidia were scored.

In the fly there are two identified *Gγ* genes^34^, one encoding a phototransduction specific form (*GγE*)^35^, leaving the other - *Gγ1*- as the likely candidate Gγ in the Go trimeric complex. Clones of *Gγ1* did not survive to allow mosaic analysis on R3/4 fates. Next, we overexpressed Gγ1 in *wild type* and *fz*^*−*^ eyes, but failed to bias the fate of R3/4 mosaics in either *wild type* or *fz*^*−*^ backgrounds (Fig. 4C). Below however, we show that Gγ1 levels become important when Gβ subunits are concomitantly overexpressed.

#### Gβ

There are 3 *Gβ* candidate genes (*Gβ76C*, *Gβ5* and *Gβ13F*) in the fly genome^34^ of which *Gβ76C* appears dedicated to phototransduction^36^. Overexpression of Gβ5 alone in *fz*^*−*^ eyes did not significantly bias R3/4 fates, but when co-expressed with Gγ1 a significant biasing to the R4 fate occurred (Fig. 4C). Overexpression of Gβ13 alone induced a significant biasing to the R4 fate; a biasing that was strongly enhanced when co-expressed with Gγ1 (Fig. 4A,C). Thus, Gβ13 appears as a more potent disturber of R3/4 fates than Gβ5 and on this basis we speculate that it is the preferential Gβ in the Go trimeric complex. A similar result occurred in our previous study of the roles of Go in asymmetric cell divisions^17^. Regardless of the exact identity of the Gβ member of this complex, the key observation is that overexpression of components of the βγ moiety act to promote the R4 fate. Thus, both the α and the βγ components of the Go trimeric complex appear to function to promote the R4 fate.

### RhoA promotes the R4 fate

Trimeric G-proteins frequently regulate small GTPases of the Rho family^37,38^ and in this regard RhoA appeared as a possible Go effector since it has been implicated in PCP signaling^19^. *RhoA* loss-of-function clones in wild-type eyes show no chirality defects^14^, but in R3/4 mosaic analyses in *fz*^*−*^ eyes, the *RhoA* mutant cell was significantly biased to the R3 fate (Fig. 4B,C). Since loss of *RhoA* drives cells to the R3 fate, we infer that the normal role of RhoA is to promote the R4 fate - the same role as Go and opposite to Dsh. Cumulatively, these results highlight the Go-Gβγ-RhoA signaling axis as antagonistic to Dsh in R3/4 cell fate choice.

### Synergy between *dsh* and *Go-Gβγ-RhoA* mutants

**Figure 5:**
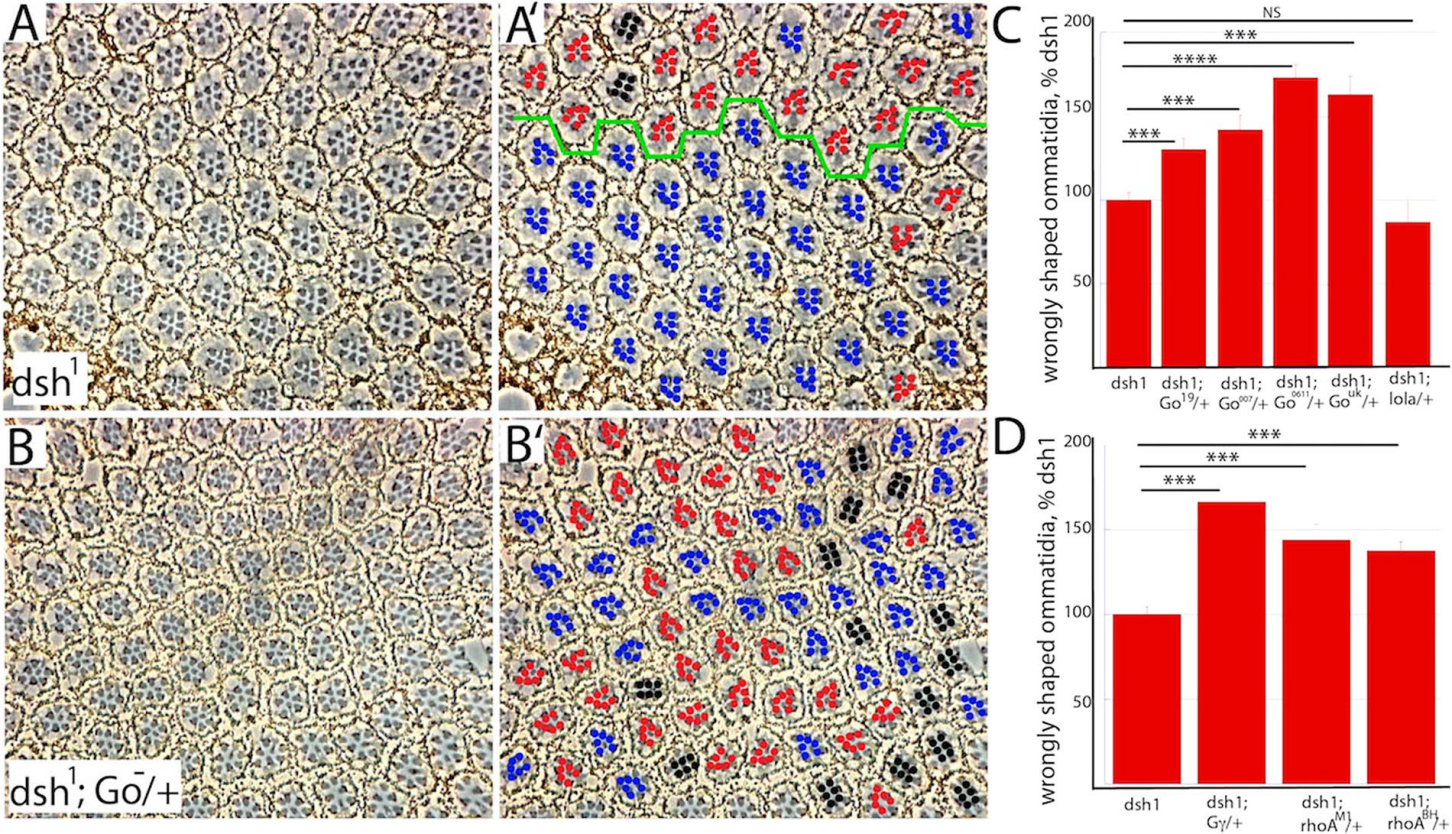
Synergistic effects of *Go* and *dsh* mutants. (**A**,A’) Only ~20% of ommatidia make the incorrect chiral choice in *dsh*^1^ eyes, and the position of the equator (green line) can often be inferred. (**B**,B’) Removal of a copy of *Go* (using the *Go*^0611^ null allele) significantly enhances the number of incorrectly chosen shapes in *dsh*^1^ eyes; no equator can be discerned. (**C**) Histogram plotting the number of incorrectly shaped *dsh*^1^ ommatidia when *Go* levels are varied. There is significant enhancement when single copies of hypomorphic *Go* alleles are introduced (*Go*^19^*, Go*^007^), and a stronger enhancement when *Go* deficiencies are introduced (*Go*^0611^, *Go*^*UK*^). Removal of one copy of the neighboring gene *lola* (removed by the *Go*^0611^and *Go*^*UK*^ deficiencies) has no effect. 6–13 eyes were analyzed for each genotype. (**D**) Histogram plotting the number of incorrectly shaped *dsh*^1^ ommatidia when *Gγ*1 or *RhoA* alleles are introduced. Both enhance the number of ommatidia which adopt the incorrect chiral shape.

The results to date had detected the actions of two antagonistic pathways; one promoting the R3 fate, and the other R4. The action of two antagonistic signaling pathways is used to sharpen polarizing information in cells undergoing chemotaxis^39^, and if something similar occurred in eye PCP, then we would expect that the effects of compromising one pathway would be enhanced by the coincident abrogation of the other. To test this, we performed experiments in the *dsh*^1^ mutant eyes (in which Dsh PCP activity is compromised but not abolished), and then modulated the levels of the other gene products in that background. In these experiments we did not perform mosaic analysis, but rather changed expression levels in a blanket manner. Thus, in this experiment the activity levels were raised or lowered in all the cells of the tissue, and we then evaluated the ability of ommatidia in that tissue to make the correct chiral choice. *dsh*^1^ mutant eyes have ~20% incorrectly shaped ommatidia (Fig. 5A); in comparison, *fz*^*−*^ mutant eyes show an apparent randomization of shapes (~50% incorrect), suggesting that *dsh*^1^ flies retain significant PCP signaling. When a hypomorphic *Go* allele (*Go*^19^ *or Go*^007^) was introduced into the *dsh*^1^ background the number of incorrect ommatidia increased significantly (Fig. 5C), and when *Go* deficiencies were introduced, an additional enhancement occurred, approaching the complete randomization found in *fz*^*−*^ eyes (Fig. 5B,C). The *lola* gene is proximate to the *Go* locus, and is removed in the two *Go* deficiencies used (*Go*^0611^ and *Go*^*uk*^), and since it regulates N/Dl interactions and is specifically implicated in R3/4 fate decisions^40^ we evaluated its ability to modulate the *dsh*^1^ chirality phenotypes and found that the number of incorrectly shaped *dsh*^1^ ommatidia did not change when a copy of *lola* was independently removed (Fig. 5C). Hence, we infer that the enhancements of *dsh*^1^ chirality defects seen with the *Go* deficiencies are not caused by the removal of *lola*.

We then evaluated whether *Gβγ* and *RhoA* gene copy reductions would modulate the *dsh*^1^ chirality defect frequency. Indeed, they did; when a copy of Gγ1 or *RhoA* were removed in the *dsh*^1^ background a significant enhancement of the chirality defects was detected (Fig. 5D). These experiments argue that both the Dsh and Go-Gβγ-RhoA activities synergize in allowing the R3/4 cells to make the appropriate cell fate choices.

### Dsh and Go/βγ antagonism in wing PCP

In this section we switch our experiments to the fly wing, for two reasons. First, the R3/4 fate biases we observed may result from effects on N/Dl interactions rather than from modulations of the upstream PCP pathway. To address this, we examined the effects of Dsh and Go/Gβγ manipulations in wing PCP in which N/Dl interactions play no part to evaluate whether the effects are likely PCP-specific. Second, the polarization mechanisms in wing and eye cells are expected to be molecularly similar, and if the interaction of the two antagonistic pathways represents a key aspect of PCP, then that interaction should also be detectable in the wing. Here we examined two distinctly different phenomena: (i) the mechanism that normally decorates each wing blade cell with a single, precisely orientated hair; (ii) the asymmetric division of the sensory organ precursor cells (SOPs) that give rise to the mechanosensory bristles.

#### Analysis of wing blade hairs

**Figure 6:**
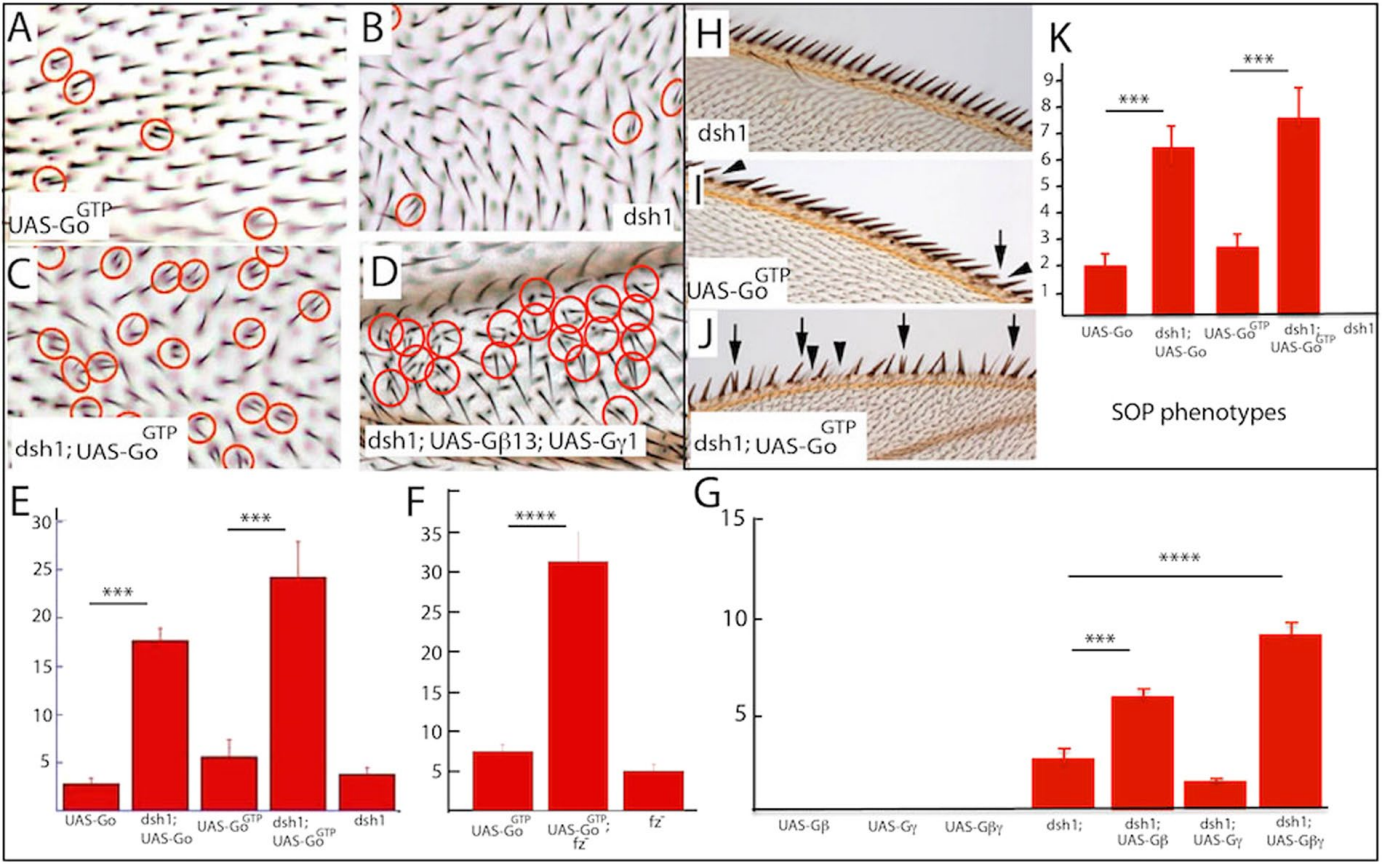
*dsh*, *Go* and *fz* genetic interactions in wing PCP. (**A**) Overexpression of *Go*^*GTP*^ in the wing induces a multiple wing hair phenotype (red circles). (**B**) In *dsh*^1^ wings multiple wing hairs occur at a low frequency (red circles). (**C**) Overexpression of *Go*^*GTP*^ in a *dsh*^1^ wing dramatically increases the number of multiple wing hairs (red circles). (**D**) There is a strong increase in multiple hairs when *Gγ*1 and *Gβ*13 are co-expressed in a *dsh*^1^ background. (**E**) Histogram plotting the frequency of multiple hairs when Go is over expressed in wild type and *dsh*^1^ backgrounds. 10 wings were examined for each genotype. (**F**) Histogram plotting the frequency of multiple hairs when Go^GTP^ is over expressed in wild type and *fz*^*−*^ backgrounds. 10–12 wings were scored for each genotype. (**G**) Histogram plotting the frequency of multiple hairs when *Gγ*1 and *Gβ*13 are overexpressed in wild type and *dsh*^1^ backgrounds. 6–37 wings were analyzed for each genotype. (**H**) *dsh*^1^ wing margins show no signs of SOP asymmetric cell division defects. (**I**) Margins from wings in which Go^GTP^ is overexpressed show loss (arrowheads) or duplication (arrow) of bristles. (**J**) When Go^GTP^ is overexpressed in *dsh*^1^ wings the margins show extensive evidence of asymmetric division defects. (**K**) Histogram plotting the frequency of margin bristle defects when Go is overexpressed in wild type and *dsh*^1^ backgrounds. 6–12 wings were scored for each genotype.

In the wing, each cell polarizes to form a single distal focus from where the hair grows out. Mutants such as *fz*^*−*^ or *dsh*^*−*^ show two distinct PCP phenotypes here. In one, the cells project their hairs in the wrong direction (the focus is incorrectly positioned), and in the other they produce more than one hair per cell (the cells produce more than one focus; the multiple wing hair phenotype). *dsh*^1^ encodes a protein deficient for PCP but able to transduce Wnt signaling^41,42^ and produces full sized wings defective only in PCP - they show the misdirection of the hairs and the multiple hair phenotypes (Fig. 6B). Previously we detected an effect of Go expression on multiple wing hair induction^15^, and we used this phenotype to assess genetic interactions between *dsh* and *Go*. In *dsh*^1^ mutants there is a modest frequency of multiple wing hairs (Fig. 6B), as there is when Go is overexpressed^15^ (Fig. 6E), but when the two were combined there was a dramatic phenotypic increase (Fig. 6E). A similar effect was detected when Go^GTP^ was overexpressed in a *wild type* (Fig. 6A,E) or *dsh*^1^ background (Fig. 6C,E). Hence, the potency of Go to induce multiple hairs is enhanced by *dsh*^1^, and by inference Dsh constrains the ability of Go to induce multiple hairs. Thus, here in the wing, as in the eye, Dsh and Go show antagonistic activities.

We previously examined the potency of Go^wild type^ and Go^GTP^ expression to induce multiple hairs in *wild type* or *fz*^*−*^ wings^15^. These experiments were designed to investigate whether Fz may act as the exchange factor for Go. We revisited this experiment in this current study and scored the numbers of multiple hairs that were formed in presence or absence of Fz. In the absence of Fz, expression of Go is unable to induce multiple wing hairs, but the effect of Go-GTP is not only maintained in the *fz* mutant background, but is dramatically enhanced (Fig. 6F). Hence when Go is in the active state (bound to GTP) and Fz is present, there is a constraint on the ability of Go-GTP to induce the multiple wing hair phenotype. But when Fz is removed, that constraint is removed, and we posit that in the absence of Fz, Dsh activity is significantly reduced and it is no longer able to restrain the effects of Go.

Next we used the multiple wing hair phenotype to look for genetic interactions between the *dsh*^1^ and Gβγ genes. Overexpression of Gγ1, Gβ13, or the combination of the two showed no phenotype (Fig. 6G), but in the *dsh*^1^ background expression of Gβ13 strongly enhanced the multiple hair phenotype, and an even more pronounced enhancement occurred when Gγ1 was co-expressed (Fig. 6D,G).

#### Analysis of SOPs

Polarization mechanism regulate the asymmetric divisions of SOPs that generate the mechanosensory bristles of the wing margin. Furthermore, overexpression of Go is known to disturb the divisions of these SOPs resulting in the loss and/or duplication of the bristles^17,43^. These Go SOP phenotypes are sensitive to Dsh activity in a similar way to that described above; the effects are markedly increased in the *dsh*^1^ background (Fig. 6H–K). Furthermore we previously documented that Go^GTP^ SOP phenotypes are not only active in a *fz*^*−*^ background, but are strongly enhanced^17^, again suggesting that the removal of Fz releases a constraint on activated Go.

Collectively these experiments satisfy the two questions that motivated the research in this section. First, the proteins associated with R3/4 fate decision are clearly implicated in polarizing mechanism in which N/Dl interactions do not operate. This bolsters the premise that the PCP pathway and not the N/Dl interactions mediate the effects of the protein manipulations. Second, the antagonistic pathways detected in the R3/4 decision were also found in wing PCP, arguing that antagonistic pathways represent a core feature of PCP.

## Discussion

The manifestation of PCP in the fly eye as the chiral shapes of the ommatidia has allowed us to detect two different signaling activities; one that promotes the R3 fate and the other that promotes R4. Since the R3/4 fates are ultimately directed by a N/Dl interaction, we see one category as diminishing N activity within a cell (and thereby potentiating the R3 fate) and the other as enhancing N and thereby promoting the R4 fate. Furthermore, we find that the two pathways, although ostensibly antagonistic, cooperate in ensuring that the cells make the correct R3/4 decision, and we suggest that the two act downstream of Fz, and that their antagonistic interactions are used to “sharpen” the polarizations within the cells (Fig. 2H).

A key feature of this eye work is the use of the *fz*^*−*^ mutant background. Since the N/Dl interactions here can be biased by only minor activity differences between the R3/4 precursors^8^, it provides a highly sensitized background for detecting proteins involved in the PCP mechanism.

This is particularly important when dealing with proteins involved in basic cell biological functions such as the organization of the cytoskeleton in which we expect redundant gene functions. Given genetic redundancy, the action of a gene may remain hidden in an otherwise wild type background, but the amplification of small activity differences that occurs in the *fz*^*−*^ mutant background can reveal its role. Furthermore, since the manipulations failed to generate PCP phenotypes in a wild type background, we suspect that one or more Gα subunits act redundantly with Go in mediating PCP signaling.

The action of two antagonistic pathways acting cooperatively in a cell polarization mechanism is well described for Dictyostelium amoebae as they chemotact in a cAMP gradient^39^. Here a G protein-coupled receptor activates two antagonistic proteins: PI-3 kinase which promotes the formation of the phosphoinositide PIP3 (at the expense of PIP2), and PTEN which hydrolyzes PIP3 back to PIP2. These two enzymes become distributed to opposite ends of the cells with PI-3 kinase localized to the leading edge, and PTEN restricted to the trailing end. The resulting higher levels of PIP3 at the leading edge promote actin polymerization to support the locomotive force^44^. Although slime mold amoebae are single motile cells, and PCP occurs in cells constrained and connected in an epithelium, we posit that the polarizations of the individual cells occurs in a similar manner. We do not suggest that phosphoinsotyl metabolism is used in PCP, rather we envision other molecular mechanisms serving analogous functions.

Consistent with the analogy to the polarization mechanism in chemotaxis, many of the PCP proteins relocalize in the polarizing cells, and Dsh localizes in the R3 precursor to the position of contact with R4^14^. We have not been able to detect any Go redistribution in the eye, but we note that the antibody is poor, and detection of the protein relocalizations is difficult in the eye. In the wing where the apical profiles of the cells are larger and protein localizations are more easily observed, Go distributes to the proximal side of the cell, opposite to distally located Dsh. Hence there is evidence that the factors which act oppositely on the N/Dl interaction segregate to opposite ends of the cells. Furthermore, an ability of Dsh to modulate N activity has been documented^45^, and a role for that asymmetric distribution in biasing the N/Dl interactions has already been suggested^14^.

Fz has long been known to promote the R3 fate, and Van Gogh (Vang, also known as Strabismus) preferentially promotes the R4 fate^46^. Both are transmembrane proteins, and Fz becomes localized on the presumptive R3 membrane abutting the Vang localized on the presumptive R4 membrane^14^. Interactions between the two proteins are then thought to stabilize each other’s polarized distribution, and strengthen the polarity information. Thus, this is another example of two different proteins with antagonistic activities (one promoting R3 and the other R4) cooperating to ensure that the correct chiral shapes are formed. It is important to stress here that this cooperation occurs through intercellular signaling. This is not what we envisage for the Dsh and Go-Gβγ-RhoA pathways. We see these as antagonizing each other within individual cells in a similar manner to that described above for Dictyostelium amoebae.

The redistribution of the PCP proteins in the polarizing cells highlights the fact that proteins may have more than one role in the process. Consider Fz, it is considered the receptor for the external gradient and the primary organizer of the cell polarities, and yet it becomes confined to the distal end of wing cells. It remains unclear whether Fz behaves identically at the two stages or whether it mediates different molecular functions.

An ostensibly paradoxical result is the ability of Go^GDP^ to promote the R4 fate in a similar manner to Go^wild type^ and Go^GTP^. Naively, we expected Go^GDP^ to act as a dominant negative form of the protein and promote the R3 fate. The Go^GDP^ form of the protein is Gαo[G203T], but this appears to maintain GTP binding (albeit as reduced efficiency) and is also able to activate Fz targets^33^. Thus, this protein clearly retains some wild type function, and this likely accounts for why it phenocopies the wild type version of the protein in the R3/4 assays. Overexpression of this protein in the wing does not phenocopy the effects of the wild type or GTP forms, but rather has no effect^15^. Thus we suspect that the *fz*^*−*^ eye provides an extremely sensitive background in which R3/4 choices can detect protein activities that otherwise remain hidden.

The major goal of this paper is to highlight the cooperative action of two antagonistic pathways in PCP. There are many genes implicated in PCP that we have not addressed here, but we note that that the types of experiments performed in our work can be effectively used to place each of these genes into either or both of the pathways, and a provide valuable information on how PCP is established in the cells.
